# Tracheal injury from dog bite in a child

**DOI:** 10.1186/s13019-023-02107-6

**Published:** 2023-01-16

**Authors:** Michiyo Miyawaki, Kazuya Ogawa, Kosuke Kamada, Takashi Karashima, Miyuki Abe, Yohei Takumi, Takafumi Hashimoto, Atsushi Osoegawa, Kenji Sugio

**Affiliations:** 1 Department of Thoracic and Breast Surgery, Oita University Faculty of Medicine, 1-1 Idaigaoka Hasama-machi, Yufu, Oita 879-5593 Japan; 2 Department of Pediatrics, Oita University Faculty of Medicine, 1-1 Idaigaoka Hasama-machi, Yufu, Oita 879-5593 Japan

**Keywords:** Tracheal injury, Dog bite, Children

## Abstract

**Background:**

Dog bites associated with the head and neck area in children are a common problem. Most of the lacerations are found in the upper lip and the nose region, and tracheal injury is rare [1]. Tracheal injury requires prompt and accurate diagnosis and treatment to rescue the patient. Especially in children, securing the airway is often more difficult than in adults because of their short neck and narrow trachea. In this report, we experienced a pediatric case of multiple dog bites with tracheal injuries in the neck.

**Case presentation:**

We report the case of a 3-year-old girl who presented with multiple dog bites. There were multiple wounds on the head, face, neck, and anterior chest, and air leakage was observed from the cervical wound at the time of transfer. It was difficult to perform oral endotracheal intubation, therefore, we extended the neck wound, probed the trachea with finger, and inserted a tracheal tube directly from the cervical wound in the emergency room. Tracheoplasty and another wound cleansing were performed in the operating room. The patient was discharged on the 18th day after surgery, without further complications.

**Conclusion:**

Tracheal injury from a dog bite is rare. It is important to prompt and accurate diagnosis and treatment. Children should be especially careful because of their short necks and narrow tracheas.

## Background

Dog bites are not uncommon among animal bites. The most common site of injury is the extremities; however, the younger the child, the more likely the face and neck to be injured. Most of the lacerations are found in the upper lip and the nose region, and tracheal injury is rare [1]. Prompt and accurate diagnosis and treatment of tracheal injuries are required to rescue the patient, because of its serious and progressive symptoms, and high mortality [2]. In this report, we experienced a pediatric case of multiple dog bites with tracheal injuries in the neck. The important points in the treatment of dog bites are rapid wound treatment and infection control, especially with wounds on the neck and face.

## Case presentation

A 3-year-old girl with no significant past medical history presented to the emergency department by air ambulance because she was bitten by a neighborhood Doberman-Boxer mix dog. She had multiple wounds on her head, face, neck, and anterior chest, and air leak was observed from the neck wound. The patient was transported to the hospital with the neck wound manually held by the emergency medicine physician.

**Fig. 1:**
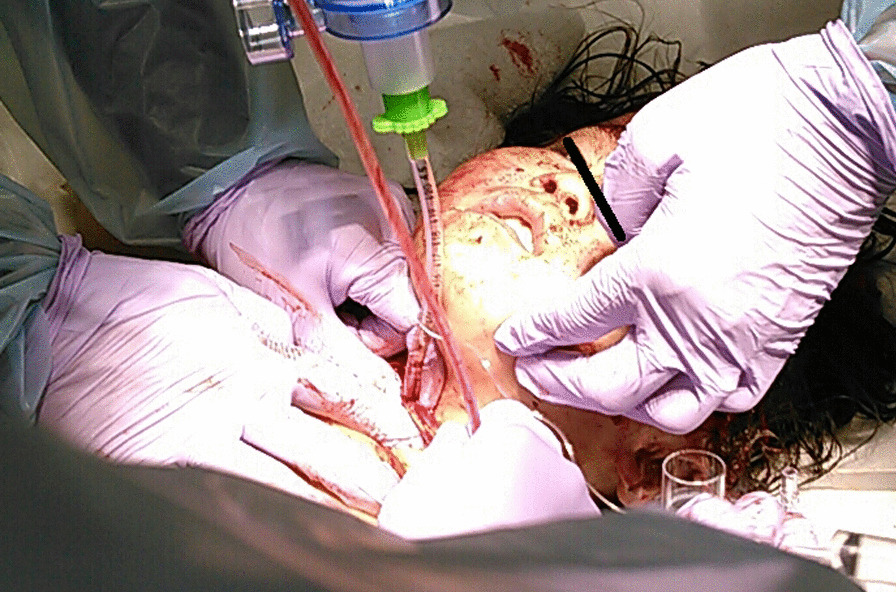
A 4 mm tracheal tube inserted directly into the trachea through the neck wound

Under sedation, blind oral endotracheal intubation was attempted by pediatrics and anesthesiology, however, the tube was trapped in the airway and could not be inserted. As ventilation became impossible, after extending the neck wound with a scalpel, the tracheal lumen was felt and confirmed with a finger, and a 4-mm tracheal tube was inserted directly from the cervical wound (Fig. 1).

**Fig. 2:**
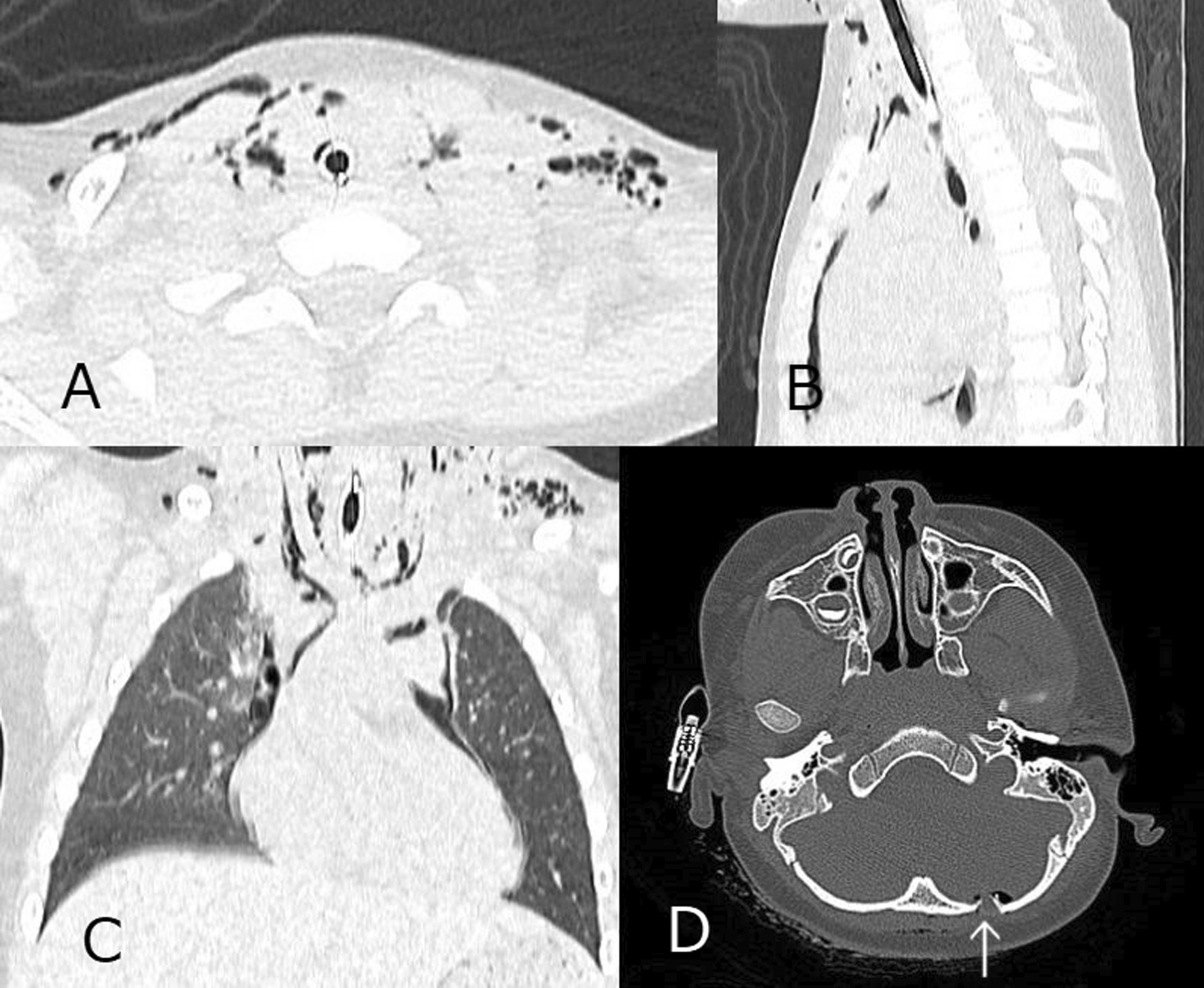
**A**, **B**, **C** Computed tomographic scan of the chest shows subcutaneous and mediastinal emphysema, but the area of tracheal injury is not clear as the tube has already been implanted. **D**Computed tomographic scan of the head shows a depressed skull fracture and a small amount of air in the intracranial space (arrow)

**Fig. 3:**
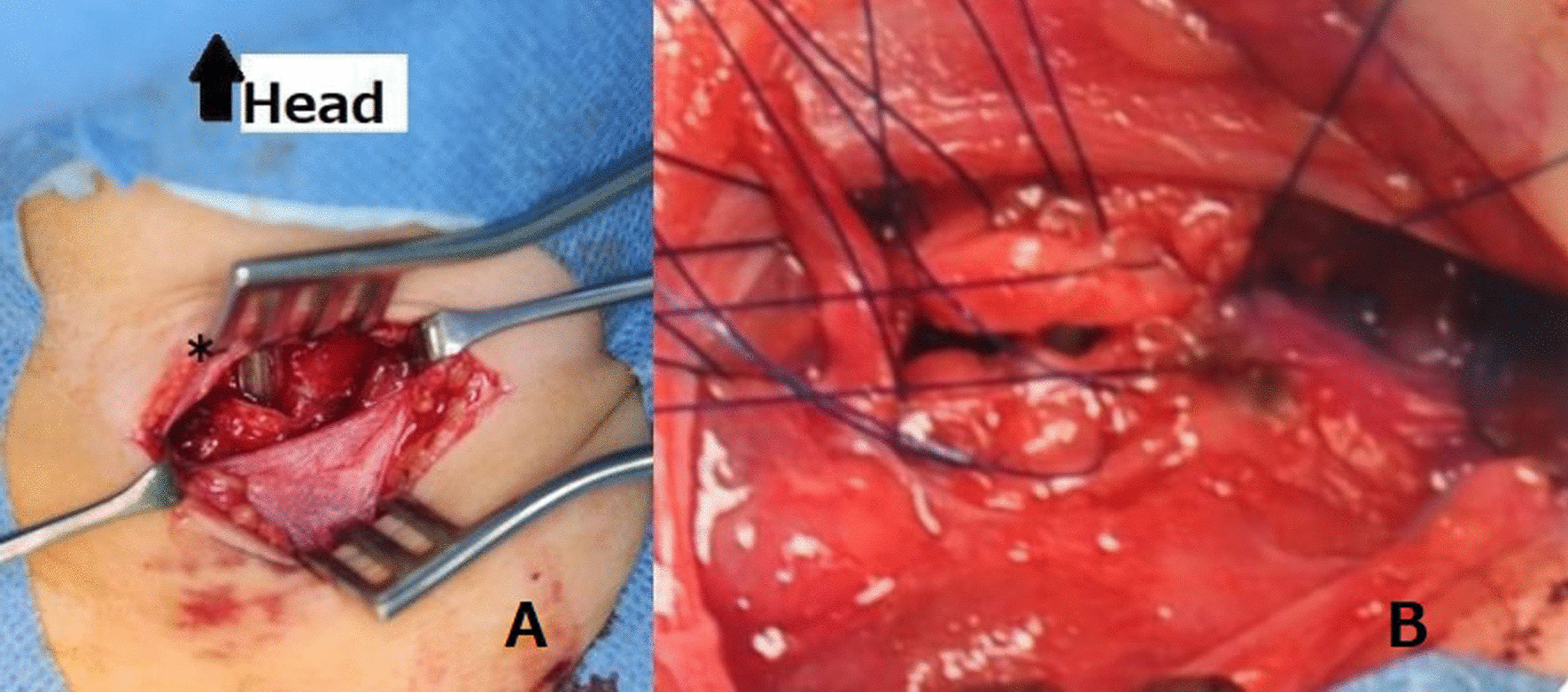
Gross examination of neck wound: **A**tracheal tube identified through neck wound (*); **B** directly anastomosed trachea

**Fig. 4:**
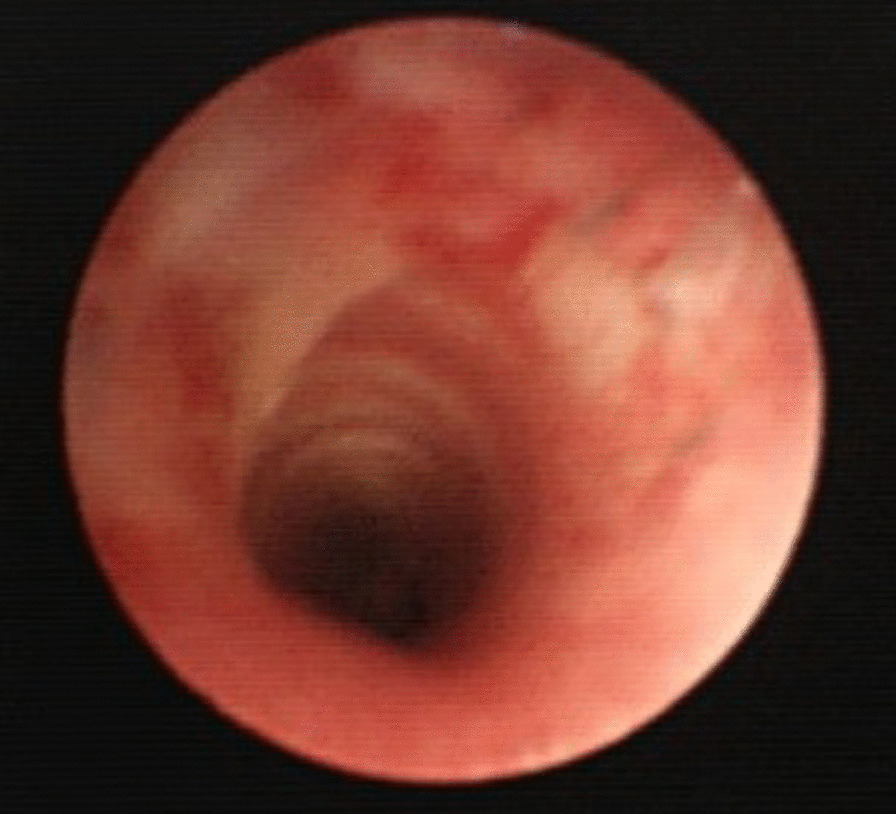
Tracheal anastomosis confirmed by bronchoscopy on postoperative day 3

The trachea was almost completely transected. An ultrathin flexible bronchoscope was prepared, after sufficient ventilation, the flexible bronchoscope was passed through a 4.5-mm intubation tube, the cervical tube was removed once, and the flexible bronchoscope was guided through the cervical wound into the lower trachea for oral intubation. After respiration and circulation had stabilized, a whole-body computed tomography (CT) scan was performed (Fig. 2). The CT scan showed not only surface wounds and subcutaneous emphysema, but also a depressed skull fracture and a small amount of air in the intracranial space (Fig. 2D). After administration of antibiotics (meropenem hydrate) and tetanus toxoid vaccine, the patient was transferred to the operating room. The tracheal tube was visible when the anterior neck wound was extracted (Fig. 3A). The space between the cricoid cartilage and the first tracheal cartilage was transected three-fifths, and almost only the tracheal membranous area retained continuity. A part of the first tracheal cartilage was damaged, but a direct suture was possible by trimming. Tracheoplasty was performed with 14 stitches using a 5−0 polypropylene suture, with simple interrupted suture (Fig. 3B). No recurrent nerves were identified. The anterior cervical wounds were closed with sutures, but the remaining multiple crushed wounds were washed with large amounts of saline and were temporarily not sutured. She was immobilized with endotracheal intubation for 3 days and was extubated after confirming that there were no problems with the tracheostomy wound by bronchoscopy (Fig. 4). The left vocal cord showed paracentral fixation, but no hoarseness of voice was observed. On postoperative day 4, secondary suture closure of the face and head wounds was performed with plastic surgery. The skull fracture was treated conservatively and antimicrobial therapy with meropenem hydrate was administered for 14 days because of pneumocephalus. The patient was discharged on the 18th day after surgery without any further wound infection.

## Discussion and conclusions

Airway trauma is a life-threatening condition that may result from blunt and penetrating neck injuries. Delayed diagnosis and treatment lead to early fatal outcomes or late sequelae, such as airway stenosis. Therefore, prompt and accurate diagnosis is mandatory for the survival of these patients. The symptoms and signs of tracheal injury depend on the site and severity of the injury; most symptoms are not specific to this type of injury. Subcutaneous emphysema is the most common finding. Other symptoms such as dyspnea, tachypnea, respiratory distress, and hemoptysis have also been observed. Additionally, air escape from penetrating neck trauma should be considered a diagnostic tool for airway injury [2].

Blind endotracheal intubation may worsen laceration and/or create a false passage for the tube. Baumgartner and associates [3] delivered this warning in 1997 in the case of a patient with complete tracheal transection with false intubation. Therefore, spontaneous breathing of the patient should be preferred until a safe airway has been achieved. As bronchoscopy represents the procedure of choice to locate the site of the injury and ensure that the tube’s cuff is inflated beyond the site of the injury, endobronchial intubation over a flexible bronchoscope is the preferred method for airway management and definitive diagnosis. However, in pediatric patients, the narrow tube limits the usefulness of flexible bronchoscopy. It was determined that this case required sedation under airway securing for a whole-body evaluation. Since pediatrics and anesthesiology were present, and assuming that the tracheal injury would be mild, oral intubation was attempted. However, the failure of intubation required an enlarged cervical wound and intubation through the neck wound. In patients with penetrating cervical tracheal injuries, tracheostomy tube insertion through the wound is the best way to secure the airway [2] [4].

Recently, the effectiveness of supraglottic devices, such as laryngeal mask airways or i-gel, has been reported for airway management during tracheal surgery [5][6]. Although not appropriate for anesthesia during surgery for tracheal lesions with air leaks, as in this case, it may have been useful for postoperative airway management because it is less irritating to the trachea and can be observed with a bronchoscope. In addition, if the airway is difficult to secure, partial support using extracorporeal life support (ECLS) should be performed as soon as possible [7].

Small lacerations of the trachea can be closed with direct sutures, while complete or partial transection requires trimming of the damaged airway edges and end-to-end anastomosis. It is important to have sufficient debridement of the damaged tracheal and bronchial cartilage to avoid postoperative complications [8]. Furthermore, early tracheoplasty should be performed before adhesion granulation occurs at the site of injury [4].

Wound management is critical in animal bites. It has been reported that with proper management (surgical grooming, edge debridement, and antibiotic prophylaxis), dog bites are less infectious and primary closure of the wound is possible [9]. This case also healed without infection with appropriate wound treatment and antibiotic prophylaxis.

The most common site of injury for a dog bite is the extremities; however, according to epidemiological data regarding pediatric facial dog bites, children aged 5 years and younger are at high risk of being bitten in the face by a familiar dog and are more likely to require hospitalization than older children. It has been reported that younger children are prone to facial injuries because: (1) they are at the same height as dogs; (2) their heads are large in relation to their bodies; and (3) they do not have the understanding or fear of dogs, and may bring their faces dangerously close them [10].

Tracheal injuries, especially in the children, are more difficult to airway management than in adults and require prompt and appropriate assessment.

## Data Availability

All data and materials are available upon reasonable request from the cor‑responding author.
